# Deep Eutectic Solvent as Green Solvent in Extraction of Biological Macromolecules: A Review

**DOI:** 10.3390/ijms23063381

**Published:** 2022-03-21

**Authors:** Jordy Kim Ung Ling, Kunn Hadinoto

**Affiliations:** School of Chemical and Biomedical Engineering, Nanyang Technological University, Singapore 637459, Singapore; jordy.ling@ntu.edu.sg

**Keywords:** carbohydrates, deep eutectic solvents, extraction, lipids, macromolecules, proteins

## Abstract

Greater awareness of environmental sustainability has driven many industries to transition from using synthetic organic solvents to greener solvents in their manufacturing. Deep eutectic solvents (DESs) have emerged as a highly promising category of green solvents with well-demonstrated and wide-ranging applications, including their use as a solvent in extraction of small-molecule bioactive compounds for food and pharmaceutical applications. The use of DES as an extraction solvent of biological macromolecules, on the other hand, has not been as extensively studied. Thereby, the feasibility of employing DES for biomacromolecule extraction has not been well elucidated. To bridge this gap, this review provides an overview of DES with an emphasis on its unique physicochemical properties that make it an attractive green solvent (e.g., non-toxicity, biodegradability, ease of preparation, renewable, tailorable properties). Recent advances in DES extraction of three classes of biomacromolecules—i.e., proteins, carbohydrates, and lipids—were discussed and future research needs were identified. The importance of DES’s properties—particularly its viscosity, polarity, molar ratio of DES components, and water addition—on the DES extraction’s performance were discussed. Not unlike the findings from DES extraction of bioactive small molecules, DES extraction of biomacromolecules was concluded to be generally superior to extraction using synthetic organic solvents.

## 1. Introduction

Extensive use of synthetic organic solvents is prevalent in petrochemical, food, pharmaceutical, cosmetic, and electronics industries for various purposes (e.g., reaction, extraction, separation, sanitization). Synthetic organic solvents exhibit great extraction capacity towards both hydrophilic and hydrophobic compounds, depending on the polarity of the solvent used [1]. A majority of these solvents are volatile organic compounds obtained from non-renewable resources (e.g., fossil fuel). While they are highly effective, synthetic organic solvents have a number of drawbacks, such as high toxicity, high flammability, and non-biodegradability [2]. The prevalent usage of synthetic organic solvents across many industries and their subsequent inevitable emissions to the environment have raised concerns over the negative long-term impacts of these solvents on the environment. Therefore, the shift towards environmentally friendly solvents (i.e., less toxic, biodegradable) has been the focus in many industries aiming to improve their manufacturing sustainability.

Several new solvents—such as subcritical water, supercritical fluids, ionic liquids, and deep eutectic solvents—have been designated as green solvents, exhibiting excellent properties, such as posing little or no toxicity to human health and the environment, use of renewable sources, reducing hazards, less energy consumption, sustainability, etc. [3]. Among the green solvents, ionic liquid and deep eutectic solvents (DES) have become increasingly popular as the two most promising green solvents [2]. Although DES and ionic liquids shared similar physical properties—such as low volatility, high viscosity, chemical and thermal stability, and non-flammability—DES is different from ionic liquid since DES is not entirely composed of ionic compound. In fact, DES can be prepared from non-ionic compounds. Additionally, compared to ionic liquid, DES is generally safer, exhibits superior biodegradability, and is more cost-effective to prepare as it is made from natural compounds [4]. At present, DESs have been used in wide-ranging applications, including electrodeposition [5,6,7,8,9], synthesis of nanomaterials [10,11,12,13], biotransformation [14,15,16,17,18,19], synthesis of pharmaceuticals and drug delivery systems [20,21,22,23,24,25], and extraction of bioactive compounds [2,26,27,28,29,30,31].

Application of DES as a green solvent in extraction of valuable compounds in particular has received increased attention in recent years, owing to its unique properties such as tailorable properties, versatility, ease of preparation, unique supermolecular structure that demonstrates high affinity towards various compounds, high solubilization power, and stabilizing ability [32,33]. A majority of studies on DES extraction, however, had focused on the extraction of bioactive small molecules [34]. A number of review articles on recent advances in DES extraction of bioactive small molecules have been reported. Interested readers can refer to the following comprehensive reviews by Fuad, Nadzir and Harun [2]; Zainal-Abidin, Hayyan, Hayyan, and Jayakumar [29]; Alam, Muhammad, Khan, Mofijur, Lv, Xiong and Xu [30]; Kalyniukova, Holuša, Musiolek, Sedlakova-Kadukova, Płotka-Wasylka, and Andruch [31]; Mehariya et al. [35]; Ali Redha [36]; Socas-Rodríguez et al. [37]; Tang et al. [38]; and Vilková et al. [39]. An example demonstrating the extractability of DES on small molecules such as phenolic compounds was reported by Bonacci et al. [40]. The authors showed that choline chloride: glycerol DES allowed high yield recovery of oleuropein (~88,287 ppm) from olive oil processing wastes after only 10 min, where the yield was found to be double of that obtained with conventional water extraction that was performed for 30 min. Similar performance of DES was also observed from the study reported by [41], where the research team found that the DES outperformed the conventional used solvents (aqueous methanol, aqueous ethanol, and water) by yielding higher recovery of polyphenols and flavonoid compounds from saffron processing wastes, once again highlighting the practicability of DES in extraction process. Despite remarkable practicability of DES extraction of bioactive small molecules, studies on DES extraction of bioactive biological macromolecules (e.g., proteins, carbohydrates, and lipids), on the other hand, had only been actively investigated more recently. Considering the unique physicochemical properties of these biomacromolecules compared to small molecules, it is of great interest to elucidate the roles of DES in extraction of biomacromolecules and the challenges faced in such applications.

In this review, we presented recent advances in DES extraction of three classes of biomacromolecules, i.e., proteins, carbohydrates, and lipids. The review began with a discussion on the green solvent characteristics of DES, followed by discussion on DES properties (i.e., viscosity and polarity) that govern its extraction performance. In the perspectives, we discussed the key findings/trends observed in DES extraction of biomacromolecules and how they were different from the findings/trends observed in DES extraction of small molecules, from which future research needs were highlighted.

## 2. Overview of DES

### 2.1. Definition of DES

DES was first proposed by Abbott et al. [42] as a sustainable solvent mixture of choline chloride with urea that, when mixed at proper molar ratio, exhibited melting points much lower than those of either individual component. The term ‘eutectic’ originates from Greek ευτηκτος, translated as ‘easy melting’, and was used by British physicist Frederick Guthrie in 1884 to describe “a lower temperature of liquefaction than that given by any proportion” [43]. Nonetheless, it is worth noting that not all eutectic mixtures can be defined as DES because essentially all mixtures of compounds that are immiscible in the solid phase present a eutectic point, as explained in detail by Martins et al. [44].

For the qualificative term ‘deep’, to-date, there is no universally agreed explanation, but most of the literature coined the term ‘deep’ as those mixtures with a eutectic temperature far below that of an ideal liquid mixture [45,46,47]. Although different denominations are used, the principle applied to classify DES is the same and the DES acronym continues to be used in an ever-broadening concept. Smith et al. [48] revisited the term ‘deep eutectic’ to include eutectic mixture of Lewis or Brønsted acids and bases which comprise a range of anionic and/or cationic species and classified them into four different types according to their constituents. Readers who are interested to know more about the details of each type/classification of DES are referred to the literature reported by Smith, Abbott, and Ryder [48]. Specifically, DES is defined as a mixture of two- or three-hydrogen-bond donor (HBD) and hydrogen bond acceptor (HBA) compounds which, at the right molar ratio, have a eutectic point temperature that is below that of an ideal liquid mixture [44,45,46,47].

### 2.2. Unique Physicochemical Characteristics of DES

**Figure 1:**
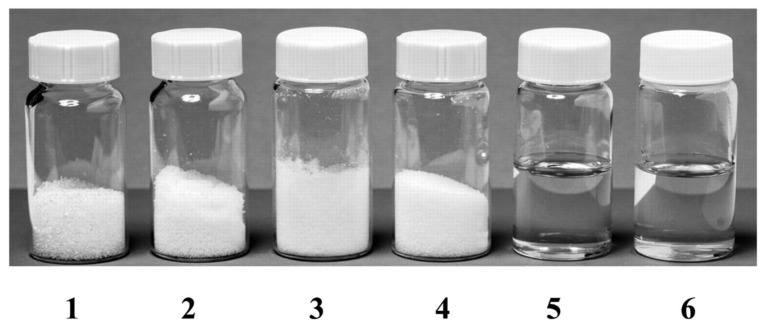
Typical natural deep eutectic solvents. Container 1, Sucrose; Container 2, Fructose; Container 3, Glucose; Container 4, Malic acid; Container 5, Sucrose: Fructose: Glucose (1:1:1, molar ratio); Container 6, Sucrose: Malic acid (1:1, molar ratio). The synthesized deep eutectic solvents (container 5 and 6) are liquid in form. Reprinted with permission from [49]. Copyright 2003 American Pharmaceutical Association.

**Figure 2:**
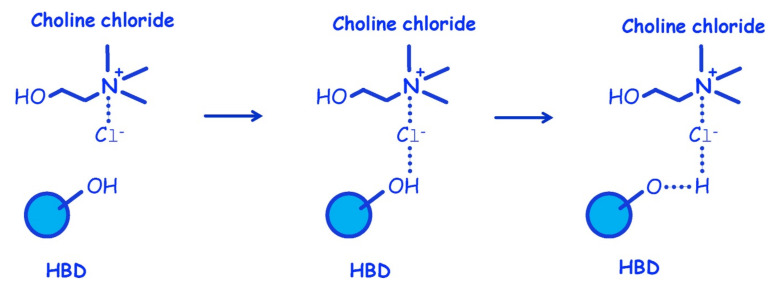
Synthesis of choline chloride-HBD DES: hydrogen bond formation. Reprinted with permission from [50].

An interesting phenomenon exhibited by DES is that pure solid components can become liquid by mixing them in a certain ratio under a mild heating. An example of this phenomenon in DES synthesis is depicted in Figure 1, showing that the sugars (sucrose, fructose, and glucose) and malic acid are solid at room temperature (melting point above 130 °C), while their combinations (denoted as DESs) are in liquid form [49]. Upon mixing, the HBD and HBA components will interact with each other through hydrogen bonding. A simple illustration of DES synthesis composed of choline chloride and HBD through hydrogen bonding interaction is shown in Figure 2. As shown in Figure 2, there is a formation of hydrogen bond interaction between the chloride ion of HBA and the OH-group of HBD [50].

The important features of DES include a lower melting point of synthesized DESs than their individual components, low cost, low toxicity or non-toxicity, renewability, sustainability, ease of preparation, and physicochemical properties of the DES which could be manipulated to suit a wide range of applications. Furthermore, DES synthesis does not involve a purification step and it is recyclable, hence reducing the costs and making it more environmentally friendly. What makes it more environmentally friendly is that DES has low vapor pressures which reduce their emission to the atmosphere [51,52]. All of these benefits have reinforced the greenness of DES, making it a suitable alternative to synthetic organic solvents.

With these remarkable advantages, numerous DES formulations have been designed and reported. In particular, the use of primary cellular constituents such as amino acids, alcohol, carboxylic acids, and sugars as HBD—first proposed by Choi, van Spronsen, Dai, Verberne, Hollmann, Arends, Witkamp, and Verpoorte [49]—has received tremendous attention. Choi and the team termed this subclass of DES as the natural deep eutectic solvents (NADESs) as they are natural, have no adverse effects, and are highly compatible with food, pharmaceutical, nutraceutical, and cosmetic formulations [53].

**Figure 3:**
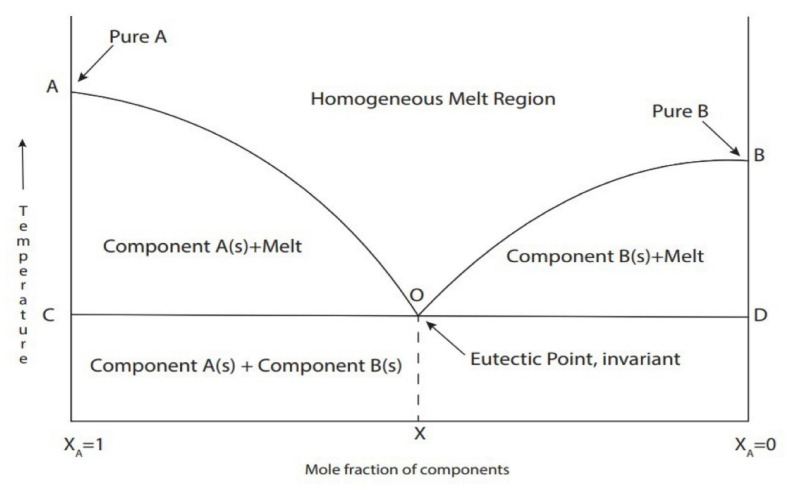
Schematic representation of the DES at the eutectic point. The solid line shows the melting temperature as a function of the mole fraction of components in the mixture and the dashed lines show the temperature and composition of the eutectic mixture. Reprinted with permission from [54].

As mentioned before, one of the unique features of DES is that it has a lower melting point than that of its individual HBD and HBA components. Lower melting points are attributed to the formation of hydrogen bond interactions between the mixture components, which decreases the lattice energy of the system and thus reduces the melting point [48]. Figure 3 shows the formation of a eutectic solution corresponding to the minimum melting temperature and it has a significantly lower melting point compared to its individual components. An example by Abbott, Capper, Davies, Rasheed, and Tambyrajah [42] demonstrated that the melting point of choline chloride:urea DES prepared at molar ratio of 1:2 is 12 °C was considerably lower than that either of the individual components: choline chloride (302 °C) and urea (133 °C).

With a low melting point, DES exists as a liquid solvent at ambient temperature and can be applied as extraction solvent. It is believed that the melting point depression depends on the lattice energy of DES and the interaction between the anion and HBD. By using choline chloride and urea as example, the melting point depression arises from the development of strong interaction between the chloride ion and urea molecules. That is the formation of hydrogen bonds between the halide anion of the salt and the HBD, where an increase in hydrogen bond interactions with anionic groups can reduce the interaction with cationic groups. As a result, this weak interaction (low lattice energy) between the anionic and cationic groups consequently results in the reduction in the melting point [29].

Another significant finding is that the molar ratio of HBA and HBD can also affect the melting point of DES as evidenced in the example of choline chloride:urea DESs—at molar ratios of 1:1 and 1:2—exhibit melting points of >50 °C and 12 °C, respectively [55]. Besides the effect of the molar ratio of HBA and HBD, the choice of HBD component also has significant influence on the resultant DES’s melting point. For instance, the use of citric acid, malonic acid, oxalic acid, glycerol, ethylene glycol, and xylitol as HBD resulted in the formation of DESs with melting temperatures of 69 °C, 10 °C, 34 °C, −40 °C, −66 °C, and room temperature, respectively, proving that the type of HBD influences the melting point of the synthesized DES [55]. More examples reporting the melting point of different combinations of DESs can be found in studies reported by Tang, An, and Row [38]; Smith, Abbott, and Ryder [48]; Zhang, Vigier, Royer and Jerome [55]; Maugeri and de María [56]; Cao and Su [57]; and Rahman et al. [58], while some of the examples from these studies are summarized and presented in Table 1.

### 2.3. DES Preparation

DES can be easily prepared by mixing the components at a temperature lower than 100 °C with agitation and a subsequent purification step is not needed, which helps to maintain a comparatively low production cost and facilitates industrial-scale applications [48]. The heating method is the most common practice since it is easier to prepare and has lower cost. Aside from the moderate heating method, other approaches—such as vacuum evaporating [33], freeze drying method [67], grinding method [68], and extruding method [69]—have also been adopted to prepare DES. For instance, Florindo, Oliveira, Rebelo, Fernandes and Marrucho [68] reported that the DES composed of cholinium chloride and glutaric acid obtained via grinding has a higher purity compared to that formed via conventional heating method. The lower purity of the DES that was obtained by using conventional heating method is likely due to the introduction of heat, which induced the formation of ester between carboxylic acid and the components containing OH group (alcohol moiety of CHCL), which subsequently affects the properties of DES [70].

Besides the aforementioned conventional ways, a greener microwave-assisted preparation approach was recently introduced by Gomez et al. [71]. Compared to the conventional heating method, the microwave-assisted method only needs 20 s of synthesis time to prepare the DES, which highlights its feasibility in preparing the DES. On the other hand, Santana et al. [72] compared different methods to prepare DES, including conventional heating method, microwave-assisted method, and ultrasound-assisted method. The research team found that there are no significant differences of physical properties among the DES produced by using different preparation methods. The differences of electricity consumptions were significant, where for heating method, microwave-assisted method and ultrasound-assisted method, the electricity consumption was 0.014, 0.106, and 0.006 $\mathrm{KWh}\;{\mathrm{mL}}^{-1}$, respectively. It is apparent that in terms of electricity consumption, the ultrasound-assisted method is more preferrable and green. Nevertheless, given the relative ease of preparation using the conventional heating method, a detailed analysis is still needed to determine the best alternative DES preparation method, mainly from the perspective of use of equipment, energy requirement, and economics evaluation.

## 3. DES as Extraction Solvent

In general, the properties of a solvent play important roles in deciding its applications. For DES, these properties include phase behavior, melting temperature, density, conductivity, surface tension, viscosity, and polarity [48,57,73,74]. For DES application as a green extraction solvent, viscosity and polarity have been identified as the two DES properties that have large influence on the extraction performance [75,76]. One of the advantages of using DES is that its properties can be easily tailored by changing the type of DES components, the molar ratio between the components, and by water addition. With tailored DES properties, high extraction efficiency can be expected and following that, the use of column chromatography with a microporous resin can be introduced to purify bioactive compounds from DES, as shown in study reported by Dai, Witkamp, Verpoorte and Choi [33].

However, a point to argue here is that the separation of bioactive compounds from DES may not be necessary, depending on the components used to synthesize the solvent and its end-application. To some, the DES containing the extracts may be directly used without solvent removal process, especially when the use of DES has enhanced the biological activity. For instance, the study reported by Ling, Chan, Nandong, Chin, and Ho [52] showed that the formulated DES enhanced the solubility and antioxidant capacity of antioxidant extracts by up to 15% and 14.64%, respectively. Within this context, it is arguable whether the solvent evaporation of DES or separation of antioxidant compounds are still necessary. It is therefore thought-provoking to further justify the needs of this practice in future studies, depending on its end-use. In this section, we discussed the effect of viscosity and polarity on the extraction performance, and also discussed how the viscosity and polarity can be tailored by the addition of water and changes in the extraction condition (e.g., temperature).

### 3.1. Viscosity of DES

The viscosity of DES is considered as one of the determining factors in influencing extraction performance. In general, most of the DESs possess a comparatively higher viscosity (>100 cP) at room temperature, which greatly limits their extraction applications. Their high viscosity reduces the mass transfer rate between the sample and extraction phase, owing to the formation of extensive hydrogen bond networks between the HBA and HBD component [39,55]. For example, Dai, Witkamp, Verpoorte, and Choi [33] used several types of DES, including 75% (*v/v*) in proline: malic acid DES in water (75% PMH), 75% (*v/v*) sucrose: choline chloride DES in water (75% SuCH) and lactic acid:glucose DES (LGH) to extract metabolites from sunflower. By using carthamin as a metabolite example, it was found that the relative extraction yields in the peak area for carthamin extraction using 75% PMH, SuCH, and LGH as solvent were 134%, 152%, and 235%, respectively. The reported viscosities of PMH, SuCH, and LGH with low water content were 251, 581 and 37 ${\mathrm{mm}}^{2}/s$, respectively [33].

Notably, the extraction yield of carthamin using LGH was 23% higher than that using conventional solvent (40% methanol). These results suggested that viscosity plays a significant role in affecting the extraction performance, such that LGH with least viscosity value gave the highest extraction yield. Specifically, they found that the cinnamic acid (small molecules) were more soluble in low-viscosity DES, with the solubility increased in the order as follows: solubility value of 128.47 m mole for 1,2-propanediol: CHCL:water DES (1:1:1) (viscosity of 33 ${\mathrm{mm}}^{2}/s$) > solubility value of 124.60 m mole for lactic acid:glucose:water (5:1:3) (viscosity of 37 ${\mathrm{mm}}^{2}/s$) > solubility value of 44.29 m mole for xylitol CHCL:water DES (1:2:3) (viscosity of 86.10 ${\mathrm{mm}}^{2}/s$) > solubility value of 40.54 m mole for glucose:CHCL:water DES (2:5:5) (viscosity of 397.40 ${\mathrm{mm}}^{2}/s$), suggesting that the viscosity of the DES affects the solubility of target samples and leads to lower extraction efficiency [33].

In addition, Xu et al. [77] also pointed out that the extraction yields of citrus flavonoids were influenced by the viscosity of DESs, where the results showed that DESs—such as CHCL:acetamide DES, CHCL:1,2-propanediol DES, CHCL:ethylene glycol DES, and CHCL:levunic acid DES—with low viscosity (<200 mPa·s, 30 °C) gave higher extraction yields (>48.17 mg/g) of total citrus flavonoids while compared to DESs—such as CHCL:xylitol DES, CHCL:sorbitol DES, CHCL:maltose DES, CHCL:tartaric acid DES, CHCL: glucose anhydrous DES, CHCL:malic acid DES, and CHCL:fructose DES—which features comparatively higher viscosity (>5000 mPa·s, 30 °C), once again confirms that the viscosity affects the extraction efficiency. Despite the effects of viscosity on extraction performance being apparent, a point to mention here is that viscosity of solvent is not the dominant parameter affecting the extraction efficiency, other extraction parameters—such as polarity, temperature, time, etc.—shall also be taken into account.

Furthermore, the changes in viscosity are related to the choice of HBD component, such that when ethylene glycol, glycerol, and phenol are used as HBD, the synthesized DES appears to be less viscous. On the other hand, the DES gives higher viscosity when sugar and carboxylic acid are employed as the HBD [38]. The trend can be observed from the examples tabulated in Table 1. When comparing viscosity of CHCL:glycerol and CHCL:ethylene glycol at molar ratio of 1:2, it was realized that the neat CHCL:glycerol DES has a relatively lower viscosity (376 cP) as compared to CHCL:ethylene glycol DES (36 cP). This is likely due to the presence of a higher number of -OH groups in the glycerol, which induces stronger hydrogen bonding interaction between the HBA and HBD components [27]. Even so, the viscosity of the polyol-based DES was comparatively lower than sugar-based and carboxylic acid-based DES, e.g., CHCL:glucose DES (9037 cP), CHCL: malonic acid DES (721 cP), CHCL:oxalic acid DES (231 cP), CHCL:ascorbic acid DES (51,570 cP), etc., as presented in Table 1.

Another point worth noting is that the temperature plays a significant role in influencing the viscosity of DES. As shown in Table 1, the CHCL:urea DES (molar ratio of 1:1) exhibited viscosity of 750 cP at room temperature and viscosity of 169 cP at 40 °C, showing that, at higher temperature, the viscosity of DES significantly reduces [55,59]. Likewise, Ribeiro, Florindo, Iff, Coelho, and Marrucho [65] also showed that the menthol:carboxylic acid (acetic acid, lactic acid, pyruvic acid, and lauric acid) DES had lower viscosity at higher temperature (shown in Table 1). A possible explanation was that, at higher temperature, the internal resistance of molecules decreased and caused the molecules flow more easily and was thus less viscous [78].

Aside from increasing the temperature, researchers also introduced water to DESs to reduce their viscosity. It was pointed out by Dai et al. [79], where the addition of water greatly reduced the viscosity of DESs as a result of the gradually weakened hydrogen bonding interaction between the DES components. Importantly, they suggested that the interaction between DES components was weakened and even disappeared when the water content was above 50% (*v/v*). That said, when excess water is added to DES, the hydrogen bond interactions between the HBA and HBD are likely to reduce and, at that stage, DES loses its unique eutectic properties and exists like a liquid with individual HBA and HBD components [52].

Therefore, the effects of water content in the DES must be studied in order to prepare DES with reduced viscosity, while at the same time, the DES is able to retain its unique eutectic properties. The possibility of adjusting the viscosity of DESs by adding water to them greatly expands their applications, predominantly in extraction processes, such that with reduced viscosity, the DES allows better solubilization of targeted extracts, enhances mass transfer rate between the sample and solvent, and results in a better extraction performance.

### 3.2. Polarity of DES

It is commonly known that the polarity of an extraction solvent is also a key factor in dissolution capacity for a desired compound. With the principle of ‘like dissolve like’, solvents with a polarity value close to the polarity of the targeted solutes presents a better solubilization capacity and allows greater extraction efficiency [80]. Similarly, the polarity of DES could greatly affect the extraction performance. The polarity of DES increases with an increasing proportion of water, which in turn can significantly affect their ability to extract target compounds. For example, Dai, van Spronsen, Witkamp, Verpoorte, and Choi [53] showed that the polarity of 1,2, propanediol-CHCL DES changed from 50.07 kcal/mol to 48.38 kcal/mol when 50% (*v/v*) of water was added, confirming the influence of water on the polarity of DES.

Another important point to take note of is that the polarity of DES varies when different HBD components are used, where organic acid-based DESs are reported to be the most polar (44.81 kcal/mol) and both sugar and polyalcohol-based DES are less polar, with a polarity value close to that of methanol (51.89 kcal/mol) [53]. Such differences in extraction efficiency can be noticed in the total anthocyanin content, with CHCL:malic acid DES being the most suitable solvent to extract anthocyanins which are polar molecules (24 mg/g dw), outperforming to those extracted by using CHCL: glucose DES (16 mg/g dw), CHCL: fructose DES (17 mg/g dw), CHCL: xylose DES (20 mg/g dw), and CHCL: glycerol DES (12 mg/g dw) [81].

Another example demonstrating the effect of polarity on extraction efficiency is shown in the study performed by Xu, Ran, Chen, Fan, Ren, and Yi [77], where the authors used different DESs with different polarity to extract flavonoids from citrus peel waste. The results showed that the extraction yields were significantly higher when DESs based on less-polar HBDs—such as acetamide, levulinic acid, and 1,2-propanediol—with CHCL: levulinic acid DES which features the lowest polarity among all of the tested DESs presenting the highest total flavonoids content (53.17 mg/g).

Another study by Tang and Row [82] prepared a hydrophilic-based DES composed of hexafluoroisopropanol and choline chloride (HFIP:CHCL), and a hydrophobic-based DES made up of menthol and tricaprylylmethylammonium chloride (Menthol: N_8881_Cl) and evaluated their performance on the extraction of high polarity (caffeic acid, chlorogenic acid, quercetin, and anthocyanidins) and low polarity (artemisinin) compounds from *Artemisia annua* leaves. Interestingly, they pointed out that alteration of mole ratio of the DES component resulted in the increment of polarity of HFIP:CHCL DES, where the order of polarity of HFIP:CHCL was 1:1 > 0.75:1 > 0.5:1. The results showed that the HFIP:CHCL at a mole ratio of 1:1 showed best extraction performance on high polarity compounds such as caffeic acid with extraction amounts of 4.75 mg, whereas HFIP:CHCL (0.75:1) was more suitable to extract polar compounds with moderately high polarity such as chlorogenic acid (4.21 mg) and caffeic acid (4.21 mg). For HFIP:CHCL (0.5:1), it was more efficient in extracting compounds with lower polarity such as quercetin and anthocyanidins, with extraction amounts of 2.97 mg and 3.66 mg, respectively, but poor performance on the others.

The effects of molar ratio on the polarity of DES and its extraction efficiency can also be observed in the case of using Menthol:N_8881_Cl hydrophobic DES as the extraction solvent. The results displayed that Menthol:N_8881_Cl at a mole ratio of 2:1 was least polar as compared to Menthol:N_8881_Cl at a mole ratio of 1:1, and 1.5:1 exhibited the best extraction performance for artemisinin (4.87 mg) but poor performance in extracting other compounds with high polarity. Their findings indicate that regulating the polarity of a solvent by tailoring the mole ratio of DES components is a viable way to manipulate the extraction performance and provides great promise in altering the DES polarity for extraction of compounds at different polarities.

From these studies, several key findings have emerged. First, the viscosity of a DES should not be too high as it affects the solubility of targeted compounds, reduces the mass transfer from samples to the solvent, and subsequently results in lower extraction efficiency. Water plays an important role as a co-solvent to reduce the viscosity and facilitates the extraction process. Second, in tailoring the DES components, mainly the HBDs can greatly affect the physicochemical properties of DES, an evaluation of the properties is therefore necessary to ensure optimized extraction performance. Third, aside from DES properties, attention should also be paid to the extraction process variables—such as extraction temperature, time, and liquid–solid ratio—as they also play critical roles in the extraction efficiency of target compounds. Fourth, the employment of advanced technology—such as ultrasound or microwave—are suggested as they improve the extraction performance. In short, the adjustable physiochemical properties of DESs, mainly viscosities and polarities, are important in extraction processes and these properties can be tailored to meet various process requirements.

## 4. DES Extraction of Biological Macromolecules

DES has played a significant role in the development of a sustainable extraction process for various bioactive compounds. Nevertheless, to-date, the applications of DES in extraction of biological macromolecules remain limited. Biomacromolecules are fundamental compounds of life and can be abundantly derived or extracted from natural sources such as plants, animals, and microorganisms [83]. Biomacromolecules, which can be broadly classified into proteins, carbohydrates, lipids, and nucleic acids, are of great interest to be exploited in various industries—including pharmaceutical, biomedical, cosmetic, and food industries—owing to their unique structural and functional characteristics [84,85]. Therefore, effective extraction of biomacromolecules from their natural sources has always been a research topic of interest. In this section, we reviewed the application of DES in biomacromolecule extraction with special attention being given to the proteins, carbohydrates, and lipids.

### 4.1. Proteins

**Figure 4:**
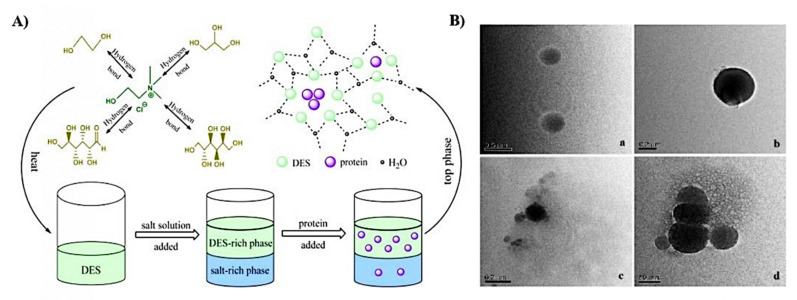
(**A**) Illustration of DES-based ATPS for the extraction of protein. (**B**) The conformation of DES and BSA solution are spherical and it is obvious that DES-proteins aggregates were formed after extraction, shown in TEM images of: (**a**) choline chloride-glycerol DES; (**b**) BSA; (**c**,**d**) BSA in DES-phase. Reprinted with permission from [88].

Proteins are macromolecules comprising linear polymers of amino acid residues joined by peptide bonds which exhibit different structures and compositions, giving different functional and nutritional properties. Proteins can be classified as globular (albumin), fibrous (collagen and keratin), or flexible (casein), depending on their structure and sequence on the polymer chain [86]. With the rising awareness of producing sustainable and high-quality proteins in diet and food formulations, development of effective extraction process for proteins is of paramount importance [87]. The extraction of proteins by using DES as an extraction solvent can be categorized into solid–liquid extraction and liquid–liquid extraction. In this context, the solid–liquid extraction is based on the dissolution of the analytes between solid samples and a liquid solvent (DES), whereas the liquid–liquid extraction refers to the partitioning of compounds of interest into one of the two immiscible phases (one being DES), as shown in Figure 4A [88,89]. The extraction of proteins by using DES via solid–liquid and liquid–liquid extractions are further discussed in this section and the examples are summarized in Table 2.

For solid–liquid extraction method, Yue, Zhu, Yi, Lan, Chen, and Rao [90] prepared nine choline chloride (CHCL) and butanediol isomer mixtures to extract oat protein and they found that the oat proteins extracted by CHCL-1,4-butanediol/water binary mixture exhibited the highest protein content (55.72%), solubility, stability, and foaming capability. Importantly, the authors pointed out that isomer of butanediol (hydrogen bond donor) is key in influencing the properties of extracted oat proteins. They also found that the proteins precipitated quickly in some DES and the DES/water binary mixture took more than 12 h for the precipitation to occur. This phenomenon might be due to the polarity of DES considering the compositional divergence of DES and less polarity being more beneficial in protein precipitation.

Chen, Chaihu, Yao, Cao, Bi, Lin, and Chen [91] used CHCL:glycerol DES as the extraction solvent to extract soy proteins and noticed that DES-extraction resulted in approximately 10% higher yield (*w/w*) as compared to the conventional alkali solution acid precipitation method. In addition, the soy proteins extracted by DES exhibit better heat resistance and stronger hydrophobicity than commercial soy proteins, indicating that DES can improve the functional properties of proteins and the extracted proteins may be used as new functional material. Besides that, Lin, Jiao, Zhang, Celli, and Brooks [92] extracted proteins from bamboo shoot by using CHCL-levulinic acid DES and compared the solvent efficiency with conventional solvent (sodium hydroxide). Under optimized conditions, roughly 60% higher protein yield (*w/w*) was extracted by using DES as compared to protein yield that was extracted by using sodium hydroxide, once again suggesting that DES exhibits great extractability for proteins.

In another study, Wahlström, Rommi, Willberg-Keyriläinen, Ercili-Cura, Holopainen-Mantila, Hiltunen, Mäkinen, Nygren, Mikkelson, and Kuutti [93] mixed carboxylate salts (potassium and sodium salts of formate and acetate) with urea to synthesize DES for the extraction of proteins from brewer spent grains. They found that sodium acetate:urea (NaOAc:urea, molar ratio of 1:2) successfully extracted 79% of protein yield (*w/w*) from brewer spent grains with more than 50% high protein content. Despite the high yield and protein component, it is important to note that some components used for synthesis of DESs, such as urea, are not favorable in the food industry. That said, replacing urea is necessary for making appealing protein products for human consumption. In this context, a design of safe, consumer-preferred DES or natural-based DES (NADES) should be encouraged for the potential application of DESs in the food industry.

Rodrigues, Leonardo, Gaspar, Roseiro, Duarte, Matias, and Paiva [94] used a betaine: propylene glycol DES at molar ratio of 1:3 to extract proteins from sardine processing residues, with higher protein yields (162.2 mg/g) as compared to those achieved by using water solvent (145.7 mg/g). Interestingly, with the use of a less polar DES, they observed an increment of hydrophobic amino acids content (alanine, isoleucine, leucine, and valine) in extracts obtained, indicating that less polar DESs facilitate the extraction of hydrophobic amino acids. Significantly, DES extract was found to exhibit significantly higher antioxidant and antimicrobial activity when compared to the water extract, with an increment of 3-fold and more than 250-fold, respectively. The protein extracts obtained using DES exhibited better scavenging potential (antioxidant potential), which correlated to the higher extraction yield. In addition, the extract obtained using DES has richer contents of hydrophobic amino acids (alanine, isoleucine, leucine, and valine), which could be the reasons for the enhanced antioxidant activity.

On the other hand, as pointed out by the authors, the positive antimicrobial activity might be attributed to the utilization of less polar DES as an extraction solvent, where it is well-established that features of peptides—such as the presence of a hydrophobic core—are crucial on facilitating the interaction of antimicrobial peptides with cell walls and membranes of microorganisms. Within this context, it can be said that since less polar DES was introduced, there was a higher selectivity for the extraction of antimicrobial ingredients, which resulted in a higher antimicrobial activity as compared to the one extracted by using water, which is hydrophilic in nature. Nevertheless, such a mechanism requires further justifications and the synergistic or additive effects of the extracts with DES would also be of a subject of interest, which merits further investigation in the future.

Another interesting study performed by Hernández-Corroto, Plaza, Marina, and García [95] demonstrated that the extraction of proteins from pomegranate peels by using CHCL:acetic acid DES-ultrasonication resulted in a higher content of protein (19.2 mg/g) as compared to pressurized liquid extraction method (9 mg/g). In addition, the hydrolysates obtained from proteins extracted with the DES presented high antihypertensive capacity, which can be attributed to higher peptide release from the DES extract. On the other hand, Liu, Yu, Ge, Bai, Li, and Fu [96] employed microwave irradiation to assist DES-extraction of proteins from pumpkin seeds. A total of 93.95% of yield (*w/w*) was obtained and the protein precipitation rate was 97.97% by using an isoelectric point-ethanol-PEG 200 DES co-precipitation method.

These studies demonstrated the feasibility of coupling DES-extraction approach with other advanced techniques, such as microwave and ultrasonication, to improve the extraction performance, where integration of these combined technique shall be comprehensively examined for future applications. Another interesting finding to highlight is the potential use of PEG-based DES in the protein extraction process since PEG has been used for the stabilization of proteins. On top of that, it is approved by the Food and Drug Administration (FDA), bringing about potential applications in vast sectors, mainly pharmaceutical and food industry [101]. Continued research should be performed to further explore the utilization of PEG-based DES in fractioning high-purity protein since PEG demonstrates high affinity to proteins and they are widely used as precipitants and crystallization agents for proteins [102,103].

Noticeably, in nearly all the studies of solid–liquid extraction process, water was added to the DES to reduce the viscosity and facilitate the extraction process. It is known that the high viscosity of DES is correlated with the presence of extensive hydrogen bonding interaction between the components of DES and the high viscosity can greatly hinder the solubilization of proteins [79]. With the addition of water to the DES, the viscosity reduces and can cause changes in the structure of DES which loosens the H-bonded structure and, as a result, it provides more spaces for the solubilization of proteins and improves the extraction efficiency. A key point to note here is the use of suitable water content because over-dilution can lead to disruption of hydrogen bonding interactions between the DES components. At that stage, the DES starts to lose its unique properties and exist like liquid with individual components [52].

In addition to the application of DESs in solid–liquid extraction of proteins, liquid–liquid extraction of protein by using DESs has also been studied. In a study performed by Xu, Wang, Huang, Li, and Wen [88], CHCL-glycerol DES was applied to form an aqueous two-phase system (ATPS) with salt solution for the extraction of bovine serum albumin (BSA) and the experimental results showed that 98.16% of BSA was extracted into the DES-rich phase, the extraction process is shown in Figure 4A. The results showed that the conformation of the protein was not changed during the extraction process as evidenced by using UV–vis, FTIR, and circular dichroism (CD) spectra. Additionally, they also stated that the separation process does not necessary rely on the electrostatic interaction, instead it is driven by the formation of protein aggregates during the extraction process, which was shown in the TEM image (presented as Figure 4B).

A similar finding was also reported in another study published by the same research group [97], confirming that DES aggregates encircle the BSA aggregate and it is the main driving force in the uptake of protein by the DES-based ATPS. The authors also highlighted that, besides the formation of aggregation, the efficiency of protein transferring to DES-rich phase is also driven by the hydrogen bonding effect, hydrophobic interactions, and the salting-out effect. Although these postulated mechanisms are helpful, more work still needs to be devoted to obtain greater understanding of the extraction chemistry and to optimize the process to ensure development of an efficient extraction method that can be up-scaled to the industrial level.

Other studies using DES-based ATPS for extraction of proteins were also reported. Xu, Wang, Chen, Wei, Xu, Ni, Meng, and Zhou [98] successfully extracted more than 98% of lysozyme into the DES-rich phase and they found that the activity of lysozyme was maintained at 91.73% after the extraction, suggesting the practicability of DES in liquid–liquid extraction method. On the other hand, Meng, Wang, Zhou, Chen, Wei, Ni, Liu, and Xu [99] used two types of DES (tetrabutylammonium chloride-polypropylene glycol 400 and L-proline-xylitol) to form ATPS for the separation of protein (chymotrypsin), where a total of 97.30% of extraction efficiency was achieved. These studies suggested potential applications of DES-based ATPS in green extraction of proteins.

However, a noticeable limitation on DES-based ATPS is the back-extraction of proteins from the DES-rich phase. Back-extraction of proteins from the DES-rich phase is very important for DES recycling and protein separation. Unlike the solid–liquid extraction process, the recovery of proteins from the DES to the aqueous solution is comparatively slow due to high interfacial mass transfer resistance. Among all these studies, only Xu, Wang, Huang, Li, and Wen [88] performed back-extraction of protein by mixing DES with fresh salt solution and manipulating the concentration of salt. As a result, 32.96% of protein was recovered. In a continued study, the same research group employed betaine-based DES ATPS for the extraction of proteins and achieved a 99.82% extraction efficiency. Unlike the former study, they introduced ethanol along with salt solution for back-extraction process in this study. In spite of that, they only managed to obtain a similar back-extraction efficiency of 32.66%, indicating that the addition of alcohol has a negligible effect [100]. In this regard, it is worth noting that the back-extraction efficiency needs to be further improved for better extraction performance.

### 4.2. Carbohydrates

Carbohydrates are essential plant macromolecules that act as building blocks for plant structures and perform functional roles in metabolic processes. In general, carbohydrates are further classified as monosaccharides, disaccharides, and polysaccharides [104]. Owing to therapeutic properties—such as anti-oxidation, anti-tumor, anti-inflammatory—and possessing a broad array of bioactivities, the extraction of carbohydrates from natural sources have gained tremendous attention [105]. Recently, the application of DESs in carbohydrate extraction has emerged, with most extraction processes focusing on polysaccharides since they are the most abundant biological materials on the planet, as presented in Table 3.

Saravana et al. [106] combined subcritical water with DES (CHCL: glycerol) to extract polysaccharide from brown seaweed (*Saccharina japonica*), where rich amount (*w/w*) of alginate (28.1%) and fucoidan (14.93%) were extracted under optimized conditions of 150 °C, 19.85 bar, 70% water content, and a liquid/solid ratio of 36.81 mL/g. It is important to note that, prior to optimization, the obtained yield by adding DES in water as catalyst was at least twice of that obtained from water/HCl as extraction solvent, suggesting that DES demonstrates an effective extraction process that improves the yield of polysaccharide.

In another study, an ultrasonication-assisted extraction of polysaccharides from another type of edible brown seaweed (*Sargassum horneri*) using CHCL: 1,2-propanediol DES was performed by Nie et al. [107]. Similarly, they investigated the optimum conditions to extract polysaccharides with high yield. The optimum extraction conditions were a molar ratio of CHCL to 1,2-propanediol of 1:2, water content of 30% (*v/v*), solid–liquid ratio of 1:30 (g/mL), and an extraction temperature of 70 °C, yielding a total of 11.31% (*w/w*) of polysaccharides. Interestingly, the authors found that when compared to conventional hot water extraction method, the polysaccharides extracted using DES had reduced amounts of proteins and minerals, indicating that DESs possess the ability to extract higher purity of polysaccharides. However, there are no other studies available to support the finding; thus, related information is still limited and shall be further explored in the future.

From these two studies [106,107], it was noticed that different combinations for DESs resulted in different polysaccharide extraction efficiencies, with CHCL:glycerol presented better extraction performance, considering the extraction yield. This might be associated to the hydrogen bonding ability and more electrostatic interactions of DES with the polysaccharides. On top of that, the occurrence of steric hindrance of three hydroxyl groups of glycerol can greatly weaken the interactions between the polysaccharide and the chloride anion, as a result, facilitating the extraction process [108]. This was also confirmed in the study reported by Saravana, Cho, Woo, and Chun [106], where they found that the CHCL:glycerol DES outperformed the CHCL:1,2-propanediol DES in extracting the alginate and fucoidan from brown seaweed. These findings reaffirmed the importance of HBD selection as it dictates the extraction performance of DES.

Cai et al. [109] designed different temperature-responsive DESs (composing of alkanolamines as HBA and cresols as HBD) to extract polysaccharides from *Ganoderma lucidum*. All the tested DES displayed higher extraction ability than water, with ethanolamine: *o*-cresol DES presented the best extractant to obtain maximum extraction yield of 92.35 mg/g and recovery yield of 88.09% under the optimum conditions of 50 wt % DES concentration, liquid–solid ratio of 30:1, extraction time of 50 min, and temperature of 60 °C. In addition, they found that the extraction yield and recovery yield of polysaccharide are stable even after the fifth cycle, with values of 81.79 mg/g and 79.31%, respectively, indicating that the synthesized DES can be recycled and reused, while maintaining high extraction efficiency. Moreover, the DES component, ethanolamine and *o*-cresol, can be easily recovered by passing through ${\mathrm{CO}}_{2}$ and $N_{2}$, reinforcing the sustainability and greenness of DES.

In a more recent study, Wu, Feng, Huang, Gan, Hu, and Zou [110] employed CHCL:ethylene glycol (1:3) DES-assisted extraction method to extract polysaccharides from lotus leaves and the extraction efficiency was compared with conventional hot water extraction method. It was found that the extraction yield of 5.38% (*w/w*) and 3.22% (*w/w*) and total polysaccharides content of 82.10% (*w/w*) and 77.94% (*w/w*) was obtained by using DES-assisted extraction and hot water extraction, respectively. A notable finding was that the extraction yield obtained by using DES-assisted extraction was higher than that of enzyme-assisted extraction (1.18% *w/w*) in a previous study [114]. The authors also found that the DES facilitated the extraction of acidic polysaccharides from lotus leaves, with higher content of total uronic acids (39.96%) being observed in polysaccharides extracted by DES as compared to the content extracted by hot water (22.98%).

Another recent study also highlighted the potential of DES in polysaccharide extraction [111]. In their study, microwave-assisted extraction coupled with the use of CHCL: 1,4-butanediol (1:5) DES resulted in the optimal extraction efficiency for polysaccharides from bladderwrack (*Fucus vesiculosus*), with extraction yields of 116.33 mg/g under following optimal conditions: water content of 32%, 168 °C, 35 min, and solid–liquid ratio of 39 mg/mL. It was apparent that, with the assistance of microwaves, higher extraction yield could be attained. This was likely due to two principal mechanisms that happened simultaneously. The first was that with rapid increase in temperature, the viscosity was reduced as well as the breakup of film and plant material, which improved the extraction rate. The second mechanism was related to molecular rotation, providing higher ion movement which leads to better extraction efficiency [115]. From their study, it showed that coupling the use of DES with other advanced extraction techniques (e.g., microwave) is a promising approach to obtain higher extraction efficiency.

Research on the utilization of DES for extraction of chitin and pectin has also been conducted. Zhu, Gu, Hong, and Lian [112] extracted high purity of chitin with a yield of approximately 20.6% (*w/w*) from lobster shell using CHCL: malonic acid DES, which is significantly higher yield when compared to that of the chemically prepared chitin (~16.5% *w/w*). Notably, the extracted chitin exhibited crystallinity up to 80.6% and presented porous structure (not shown in commercial chitin), making the extracted chitin suitable for use as adsorption material and a formative agent for tissue engineering. In addition, Shafie, Yusof, and Gan [113] performed an optimization study to extract pectin from the pickle tree (*Averrhoa bilimbi*) using CHCL: citric acid DES. Under optimized conditions of 80 °C, 2.5 h, percentage DES of 3.74% (*w/v*) and molar ratio of CHCL:citric acid components of 1:1, the extraction yield of pectin was 14.44%. It is important to mention that the extracted pectin by using DES exhibits good functional properties such as water holding capacity of 3.70 g/g, oil holding capacity of 2.40 g/g, and foaming capacity of 133.33%, making it a suitable natural biopolymer or functional food ingredient. Importantly, the use of DES was found not to jeopardize the functional properties of the chitin extracts, thus DES extraction holds great promise for commercial applications.

### 4.3. Lipids

Lipids are a diverse group of chemical compounds that can be broadly classified into two types, namely membrane lipids (polar) and reverse lipids (neutral and nonpolar). Some common polar lipids are phospholipids and glycolipids, where examples of neutral and nonpolar lipids include triacylglycerols, carotenoids, glycerides, sterols, etc. [116]. Currently, lipophilic metabolites are still extracted using solvents such as mixtures of chloroform/methanol or hexane/methanol, which are toxic and not environmentally sustainable. Therefore, research efforts to search for greener alternative solvents—such as DESs—to extract bioactive lipids and lipophilic metabolites have gained traction recently as tabulated in Table 4.

Koutsoukos, Tsiaka, Tzani, Zoumpoulakis, and Detsi [117] found that the extraction yield of carotenoids from apricot pulp using CHCL:tartaric acid DES as solvent was significantly higher in both the ultrasound-assisted extraction (4-times higher) and microwave-assisted extraction method (3.5-times higher) as compared to the extraction using conventional organic solvent, highlighting the strong affinity between DES and the lipid-rich natural metabolites. The efficiency of DES in extracting carotenoids was also confirmed in another recent study, where the authors used a series of fatty acid-based DES, combined with ultrasonication to extract β-carotene from pumpkin [118]. Under optimum conditions of 50 °C, ultrasonic power of 60% (52.5 $W/{\mathrm{cm}}^{2}$), and solvent-to-solid ratio of 7 mL/g, the highest extraction content of 151.41 μg/mL was obtained when caprylic acid:capric acid (C8:C10) DES at a molar ratio of 3:1 was used as the extraction solvent. Interestingly, the carotenoid extracts presented high stability when kept in the dark, with a total reduction of 7.3% during the storage period of 180 days, showing that DES had the capability to extend the shelf life of extracts.

In another study, three hydrophobic-based deep eutectic solvent based on oleic acid and terpenes (menthol, geraniol, and thymol) were synthesized to extract astaxanthin (carotenoid pigment) from the microalgae *Haematococcus pluvialis* [119]. All the tested DESs attained an approximate 60% recovery of astaxanthin, with thymol:oleic acid DES displayed the best extraction performances (83%) after 24 h, probably due to its high affinity towards astaxanthin. Notably, the extracted astaxanthin by using thymol:oleic acid exhibit excellent stability, with 40% of the initial astaxanthin content being maintained after 13.5 h while all other samples showed a complete astaxanthin degradation. This finding could be attributed to the higher antioxidant activity of thymol than those of geraniol and menthol. In addition, the DES components used in this study were edible and Generally Recognized as Safe (GRAS), reinforcing the suitability of DES technology for applications in the food industry as carrier or stabilizing agents for natural astaxanthin.

Shen, Wang, Zhu, Jiao, Bao, Kou, Pan, Li, and Fu [120] applied P-toluenesulfonic acid (PTSA)-based DES to produce biodiesel from seed of yellow horn (*Xanthoceras sorbifolia* Bunge) by a simultaneous extraction and transesterification process. Results indicated that by using PTSA-based DES, a total of 90.33% (*w/w*) oil extraction yield and 96.53% of fatty acid methyl esters (FAME) conversion yield was achieved at temperature of 72 °C, DES amount of 11 *wt* %, microwave power of 500 W, 40 min, and a liquid-to-solid weight of 27:1. Such a high extraction effect could be attributed to the utilization of PTSA-based DES, where the PTSA-based DES exhibits the ability to destroy the cell walls of seeds which facilitate the oil extraction process. With high extraction yield and high catalytic activity, the authors successfully produced a high-quality biodiesel that complied with the EN14214 standards, opening a path for new possible industrial applications.

The affinity of DES towards lipids was also clearly observed from the study reported by Li, Wang, Wu, Cheng, Chen, and Qi [121], where the authors proposed a deterpenation process (separation between terpenes and terpenoids), aiming to selectively extract terpenoids (linalool) from citrus essential oils by using in situ formation of a DES between a quaternary ammonium salt (tetrabutylammonium chloride) and the compound of interest (terpenoids), with an organic salt in associative extraction and recovery of terpenoids by a two-step re-extraction step using n-hexane and water. Importantly, the proposed process resulted in excellent extraction efficiency, with a recovery ratio of 89.25% and high linalool purity of 98.76%, demonstrating high affinity of DES towards lipid compounds.

Wils, Leman-Loubière, Bellin, Clément-Larosière, Pinault, Chevalier, Enguehard-Gueiffier, Bodet, and Boudesocque-Delaye [122] screened different DES combinations with a wide range of polarities for extraction of free fatty acids from spirulina. The authors pointed out that the free fatty acid-based DES improved the extraction capacity towards free fatty acids as compared to conventional reference solvent (ethyl acetate and dimethylcarbonate), with nonanoic acid:decanoic acid:lauric acid (C9:C10:C12, 3:2:1) DES demonstrated the highest selectivity and extraction performance with extract yield of 58 mg/g of formulation after intensification of the extraction process. The obtained lipid rate was 6 times better than those of other tested hydrophobic DES (octanoic acid:lauric acid DES and menthol:levulinic acid DES). The finding is reasonable since the free fatty acid profile of C9:C10:C12 extracts was dominated by saturated free fatty acids (almost 80%), allowing reliable lipid extraction. All these studies confirmed the extractability of DES for lipids.

## 5. Conclusions

Similar to the findings in the extraction of bioactive small molecules, the use of DES as a substitute for synthetic organic solvents in extraction of biomacromolecules resulted in increased extraction efficiency as characterized by higher yields. The extraction efficiency is governed by the type of DES used, particularly, the molar ratio of HBD to HBA and viscosity. By tailoring the DES properties, in some cases, the extracts obtained by DES exhibit enhanced functional properties as compared to the extracts obtained by synthetic organic solvent. In short, DES exhibits high affinity and selectivity towards biomacromolecules, making them a suitable replacement for synthetic organic solvent with great promise for commercial applications. Having said that, a number of issues remain to be addressed in future studies to realize commercial applications of DES as a biomacromolecule extraction solvent:

For food and pharmaceutical applications, the selection of DES components ideally should satisfy the GRAS requirements of the FDA. In this context, it is arguable whether the solvent evaporation of DES or separation of antioxidant compounds are still necessary. Further studies are required for further justification, depending on its end-use. Moreover, cytotoxicity studies are needed to understand the relationship between DES components and their toxicity.

The amount of water added to DES plays an important factor in affecting the extraction process. Thus, the amount of water addition needs to be controlled and optimized. On top of that, it is interesting to discuss in-depth the influence of water on the structure, polarity, and viscosity of DES in biomacromolecules extraction process, which provides insights into the underlying extraction mechanism by using DES.

For liquid–liquid extraction of proteins, back-extraction efficiency needs to be further improved for better extraction performance, perhaps a judicious choice of the DES-based ATPS forming components, salt composition, and pH need to be comprehensively examined in future studies to achieve better back-extraction efficiency. It is also important to bear in mind that the precipitation of proteins is easier in less polar DES.

The use of DES may alter the physicochemical properties of macromolecules and create new functional materials or ingredients. Therefore, it is important to examine the functional properties of the biomacromolecules after DES extraction.

DES can be recovered and reused. However, the use of recovered and reused DES for extraction beyond the laboratory scale has not been fully examined.

Recently, DES has been combined with advance extraction technologies, such as ultrasound and microwave, in extracting the target compounds. Comparative study among the different advanced DES methods is still lacking and should be taken into account in future studies. Particularly, evaluation of the energy consumption and costs is desired to better compare their feasibility.

## Figures and Tables

**Table 1:** Melting temperature and viscosity of selected DES.

HBA	HBD	Molar Ratio (HBA:HBD)	Melting Point (°C)	Viscosity (cP)	References
CHCL	Urea	1:2	12 ^1^	750 (25 °C) ^1^ 169 (40 °C) ^2^	^1^ [55] ^2^ [59]
CHCL	Ethylene glycol	1:2	−66 ^1^	36 (20 °C) ^2^	^1^ [55] ^2^ [60]
CHCL	Ethylene glycol	1:3	ND	19 (20 °C) ^2^	
CHCL	Glycerol	1:2	−40 ^1^	376 (20 °C) ^2^	
CHCL	Glycerol	1:3	ND	450 (20 °C) ^2^	
CHCL	Glycerol	1:4	ND	503 (20 °C) ^2^	
CHCL	1,4Butanediol	1:3	ND	140 (20 °C) ^2^	
CHCL	1,4Butanediol	1:4	ND	88 (20 °C) ^2^	
CHCL	Malonic acid	1:1	10 ^1^	721 (25 °C) ^2^	^1^ [61] ^2^ [48]
CHCL	Citric acid	1:1	69	ND	[61]
CHCL	Oxalic acid	1:1	34 ^1^	231 (25 °C) ^2^	^1^ [55] ^2^ [62]
CHCL	Gallic acid	1:0.5	77	ND	[56]
CHCL	Ascorbic acid	2:1	ND	51,570 (25 °C)	[52]
CHCL	Glucose	1:1	31	9037 (25 °C)	[63]
CHCL	Glucose	2:1	15	8045 (25 °C)	[56]
CHCL	Xylitol	1:1	Liquid at 25 °C	5230 (30 °C)	
CHCL	Sorbitol	1:1	Liquid at 25 °C	12,730 (30 °C)	
Thymol	Camphor	1:1	−44	25.8 (25 °C)	[64]
Thymol	10-Undecylenic acid	1:1	11	13.2 (25 °C)	
Thymol	Decanoic acid	1:1	17	11.2 (25 °C)	
Menthol	Acetic acid	1:1	−7.81	8.69 (25 °C) 3.25 (50 °C)	[65]
Menthol	Lactic acid	1:2	−61.14	218.93 (25 °C) 29.47 (50 °C)	
Menthol	Pyruvic acid	1:2	−6.78	29.95 (25 °C) 7.51 (50 °C)	
Menthol	Lauric acid	2:1	13.84	24.42 (25 °C) 7.61 (50 °C)	
Betaine	Hexafluoro-isopropanol	1:2	−39.4	76 (25 °C)	[66]
Betaine	Hexafluoro-isopropanol	1:3	−34.7	46 (25 °C)	
L-carnitine	Hexafluoro-isopropanol	1:2	−18.7	698 (25 °C)	
L-carnitine	Hexafluoro-isopropanol	1:3	−17.2	149 (25 °C)	

Note: CHCL—choline chloride; ND—not determined.

**Table 2:** Summary of the application of DES in extraction of proteins.

DES	Sample Extract	Operating Conditions	Findings	Reference
Protein (solid–liquid extraction)				
Choline chloride-butanediol	Oat proteins	Extraction temperature: 80 °C Time: 90 min	- A total of 55.72% protein content was recovered. - The oat proteins extracted by DES have high protein content, solubility, foaming capacity, and stability.	[90]
Choline chloride-glycerol	Soy proteins	Extraction temperature: 60 °C Liquid/solid ratio: 10.3 Stirring speed: 873 rpm Time: 3.9 h Water content: <15 wt %	- Higher protein yield (0.3462 g) was obtained as compared to the conventional alkali solution acid precipitation method (0.3192 g). - The soy protein extracted by DES showed better heat resistance and stronger hydrophobicity than commercial soy proteins.	[91]
Choline chloride-levulinic acid	Bamboo shoot	Extraction temperature: 80 °C Liquid/solid ratio: 30 mg/mL Water content: 40% *v/v* Time: 50 min	39.16 mg/g protein extraction yield was obtained, significantly higher as compared to conventional extraction method using sodium hydroxide (23.88 mg).	[92]
Carboxylate salt-urea	Proteins from brewer spent grains	Extraction temperature: 80 °C Time: 4 h Water content: 10 wt %	79% extraction yield (*w/w*) with >50% protein content was obtained.	[93]
Betaine-propylene glycol (B: PG)	Proteins from sardine processing residues	Extraction temperature: 80 °C Molar ratio of B:PG: 1:3 Solid/liquid ratio: 1:80 g/g Time: 18 h	- 162.2 mg/g protein yield was obtained. - The extracts increased the antioxidant and antimicrobial activity by 3-fold and more than 250-fold, respectively, when compared with water.	[94]
Choline chloride-acetic acid (CHCL: AA)	Proteins from pomegranate peels	Molar ratio of CHCL:AA: 1:2 Water content (molar ratio): 15 Amplitude: 60% Time: 15 min	- 19.2 mg/g of protein was obtained. - The hydrolyse obtained from proteins extracted by DES presented high antihypertensive capacity.	[95]
Choline chloride-polyethylene glycol (PEG)	Pumpkin seed protein	Extraction temperature: 43 °C Liquid/solid ratio: 28 g/mL Microwave power: 140 W DES concentration: 28% *w/w*	The extraction yield was 93.95% (*w/w*) (extraction was assisted by microwave irradiation) and the precipitation rate of pumpkin seed protein was 97.97, with a precipitation time of only 4 min by using isoelectric point-ethanol-PEG 200 DES ternary coprecipitation method.	[96]
Protein (liquid–liquid extraction)				
Choline chloride-glycerol	Bovine serum albumin (BSA)	Amount of DES: 1.3 g Concentration of salt solution: 0.9 g/mL Temperature: 30 °C	- 98.16% of BSA was extracted into the DES-rich phase of ATPS. - 32.96% of back-extraction efficiency was achieved.	[88]
Choline chloride-urea, tetramethylammonium chloride-urea, tetrapropylammonium bromide-urea, choline chloride-methylurea	Bovine serum albumin (BSA)	Amount of DES: 1.4 g Concentration of salt solution: 0.6 g/mL Temperature: 40 °C	The extraction efficiency was in the range of 99.94–100.05%.	[97]
Tetrabutylammonium bromide-glycolic acid	Lysozyme from chicken egg white	Amount of DES: <1.0 g Amount of salt: <0.25 g Temperature: 35 °C	- >98% of lysozyme was extracted into the DES-rich phase. - 91.73% of initial activity of lysozyme was retained after extraction.	[98]
Tetrabutylammonium chloride-polypropylene glycol 400/L-proline-xylitol [TBAC][PPG400]/[Pro][Xyl]	Chymotrypsin	Amount of [TBAC][PPG400]: 1.0 g Amount of [Pro][Xyl]: 1.6 g Amount of protein: 8 mg Temperature: 35 °C	97.30% of extraction efficiency was achieved.	[99]
Betaine-urea	Bovine serum albumin (BSA)	Amount of DES: 1.4 g Concentration of salt solution: 0.75 g/mL Amount of protein: 15 mg Temperature: 30 °C	- 98.29% extraction efficiency was achieved. - Back-extraction efficiency was 32.66%.	[100]

**Table 3:** Summary of the application of DES in extraction of carbohydrates.

DES	Sample Extract	Operating Conditions	Findings	Reference
Choline chloride-glycerol	Alginate and fucoidan from brown seaweed (*Saccharina japonica*)	Temperature: 150 °C Pressure: 19.85 bar Water content: 70% Liquid/solid ratio: 36.81 mL/g	28.1% of alginate and 14.93% of fucoidan was obtained.	[106]
Choline chloride-1,2-propanediol	Polysaccharide from brown seaweed (*Sargassum horneri*)	Molar ratio of CHCL:1,2-propanediol: 1:2 Water content: 30% (*v/v*) Solid–liquid ratio: 1:30 (g/mL) Temperature: 70 °C	- 11.31% of polysaccharide was obtained. - The polysaccharides extracted by using DES have reduced amounts of proteins and minerals.	[107]
Ethanolamine: *o*-creso	Polysaccharide from *Ganoderma lucidum*	Concentration of DES: 50 wt % Liquid-solid ratio: 30:1 Time: 50 min Temperature: 60 °C	- A total of 92.35 mg/g and recovery yield of 88.09% was obtained. - Maintained high extraction yield of 81.79 mg/g and recovery yield of 79.31% even after the fifth cycle.	[109]
Choline chloride: ethylene glycol	Polysaccharide from lotus leaves	Water content in DES: 61% Temperature: 92 °C Liquid-solid ratio: 31 mL/g Time: 126 min	- 5.38% of extraction yield and 82.10 of total polysaccharide content was obtained. - High content of total uronic acids (39.96%) was obtained.	[110]
Choline chloride: 1,4-butanediol	Polysaccharide from bladderwrack (*Fucus vesiculosus*)	Water content in DES: 32% Temperature: 168 °C Solid–liquid ratio: 39 mL/g Time: 35 min	116.33 mg/g extraction yield was attained.	[111]
Choline chloride: malonic acid	Chitin from lobster shell	Temperature: 50 °C Time: 2 h Percentage of lobster shells and DES by weight: 7%	- Approximately 20.6% extraction yield was obtained. - Extracted chitin exhibit crystallinity up to 80.6% and showed porous structure.	[112]
Choline chloride: citric acid	Pectin from *Averrhoa bilimbi*	Temperature: 80 °C Time: 2.5 h Percentage of DES: 3.74% (*w/v*) Molar ratio of DES components: 1:1	- 14.44% of extraction yield was obtained. - Extracts contain good functional properties: water holding capacity (3.70 g/g), oil holding capacity (2.40 g/g) and foaming capacity (133.33%).	[113]

**Table 4:** Summary of the application of DES in extraction of lipids.

DES	Sample Extract	Operating Conditions	Findings	Reference
Choline chloride: tartaric acid	Carotenoids from apricot pulps	Ultrasound assisted extraction: Time: 10 min, Power: 600 W Liquid to solid ratio: 35 mL/g Temperature: 30–35 °C Microwave assisted extraction: Time: 20 min, Power: 120 W Liquid to solid ratio: 45 mL/g Temperature: 70 °C	- 41.3 mg/g of β-carotene was obtained using ultrasound assisted extraction-DES, 4-times higher as compared to ultrasound assisted extraction-chloroform-methanol (11.51 mg/g). - 76.11 mg/g of β-carotene was obtained using microwave assisted extraction-DES, 3.5-times higher as compared to microwave assisted extraction-ethanol (26.50 mg/g).	[117]
Caprylic acid: capric acid (C8:C10)	Carotenoids from pumpkin	Molar ratio of C8: C10 DES: 3:1 Temperature: 50 °C Time: 10 min Ultrasonic power: 60% (52.5 $W/{\mathrm{cm}}^{3}$) Solvent to solid ratio: 7 mL/g	- 151.41 μg/mL of β-carotene content was obtained. - Extracts presented high stability during the period of 180 storage days.	[118]
Oleic acid:thymol	Astaxanthin from microalgae *Haematococcus pluvialis*	Molar ratio of DES: 1:1 Temperature: 60 °C Time: 6 h	- Around 60% of astaxanthin recovered and a total of 83% of astaxanthin recovered after 24 h. - 40% of the initial astaxanthin content was maintained after 13.5 h of light exposure.	[119]
*p*-toluenesulfonic acid (PTSA) and tetrabutylammonium bromide	Yellow horn seed oil (*Xanthoceras sorbifolia* Bunge)	Temperature: 72 °C DES amount: 11 wt % Microwave power: 500 W Time: 40 min Liquid to solid weight: 27:1	90.33% oil extraction yield and 96.53% of fatty acid methyl esters (FAME) conversion yield was achieved.	[120]
Tetrabutylammonium chloride (TBAC):linalool	Terpenoids (linalool) from citrus essential oil	Associative extraction: Mass ratio of TBAC: linalool: 20:1 Stirring temperature: 65 °C Settling temperature: 30 °C 2-step reextraction step: First step: Stirring temperature: 30 °C Settling temperature: 30 °C Second step: Stirring temperature: 25 °C Settling temperature: 25 °C	Linalool with high purity of 98.6% and recovery ratio of 89.25% was achieved.	[121]
Nonanoic acid: decanoic acid: lauric acid (C9:C10:C12)	Free fatty acids from spirulina	Molar ratio of C9:C10:C12: 3:2:1 Time: 30 min Biomass to liquid ratio: 1:20 (*w/w*)	58 mg of extraction fraction/g of formulation was obtained, with free fatty acid profile being dominated by saturated free fatty acid (almost 80%)	[122]

## Data Availability

Not Applicable.

## Abbreviations

ATPS	Aqueous two-phase system
BSA	Bovine serum albumin
CD	Circular dichroism spectra
CHCL	Choline chloride
DES	Deep eutectic solvent
FAME	Fatty acid methyl esters
FDA	Food and Drug Administration
FTIR	Fourier transform infrared spectroscopy
GRAS	Generally Recognized As Safe
HBA	Hydrogen bond acceptor
HBD	Hydrogen bond donor
HCl	Hydrochloric acid
HFIP	Hexafluoroisopropanol
LGH	Lactic acid:glucose deep eutectic solvent
NADES	Natural deep eutectic solvent
NaOAc	Sodium acetate
N_8881_Cl	Tricaprylylmethylammonium chloride
PEG	Polyethylene glycol
PMH	Proline:malic acid deep eutectic solvent
PTSA	P-toluenesulfonic acid
SuCH	Sucrose:choline chloride deep eutectic solvent
UV–vis	Ultraviolet–visible spectroscopy
